# Treatment with XAV-939 prevents *in vitro* calcification of human valvular interstitial cells

**DOI:** 10.1371/journal.pone.0208774

**Published:** 2018-12-07

**Authors:** Claudia Dittfeld, Gabriel Reimann, Alice Mieting, Petra Büttner, Anett Jannasch, Katrin Plötze, Gerald Steiner, Sems Malte Tugtekin, Klaus Matschke

**Affiliations:** 1 Technische Universität Dresden, Faculty of Medicine Carl Gustav Carus, Department of Cardiac Surgery, Herzzentrum Dresden, Dresden, Germany; 2 Technische Universität Dresden, Faculty of Medicine Carl Gustav Carus, Clinical Sensoring and Monitoring, Dresden, Germany; University of Pecs Medical School, HUNGARY

## Abstract

The development of a substance or inhibitor-based treatment strategy for the prevention of aortic valve stenosis is a challenge and a main focus of medical research in this area. One strategy may be to use the tankyrase inhibitor XAV-939, which leads to Axin stabilisation and subsequent destruction of the β-catenin complex and dephosphorylation of β-catenin. The dephosphorylated active form of β-catenin (non-phospho-β-catenin) then promotes nuclear transcription that leads to osteogenesis. The aims of the present study were to develop an experimental system for inducing *in vitro* calcification of human aortic valvular interstitial cells (VICs) to investigate the potential anti-calcific effect of XAV-939 and to analyse expression of the Wnt signalling proteins and Sox9, a chondrogenesis regulator, in this model. Calcification of human VIC cultures was induced by cultivation in an osteogenic medium and the effect of co-incubation with 1μM XAV-939 was monitored. Calcification was quantified when mineral deposits were visible in culture and was histologically verified by von Kossa or Alizarin red staining and by IR-spectroscopy. Protein expression of alkaline phosphatase, Axin, β-catenin and Sox9 were quantified by western blotting. In 58% of the VIC preparations, calcification was induced in an osteogenic culture medium and was accompanied by upregulation of alkaline phosphatase. The calcification induction was prevented by the XAV-939 co-treatment and the alkaline phosphatase upregulation was suppressed. As expected, Axin was upregulated, but the levels of active non-phospho-β-catenin were also enhanced. Sox9 was induced during XAV-939 treatment but apparently not as a result of downregulation of β-catenin signalling. XAV-939 was therefore able to prevent calcification of human VIC cultures, and XAV-939 treatment was accompanied by upregulation of active non-phospho-β-catenin. Although XAV-939 does not downregulate active β-catenin, treatment with XAV-939 results in Sox9 upregulation that may prevent the calcification process.

## Introduction

In developed countries, calcific aortic valve (AV) stenosis occurs in 1.7% of the population older than 65 years [1–4]. Development of a substance or inhibitor-based treatment or prevention strategy for this disorder is therefore a main research focus and a challenge for medical scientists [4, 5]. Calcific AV stenosis shares similar risk factors with aortic sclerosis, but its disease patterns differ; consequently, the hypothesis that aortic stenosis (AS) treatment with substances such as statins also affects calcific AV stenosis has not been confirmed [4, 6]. However, the acceptance of calcific aortic valve disease (CAVD) as an actively regulated cellular process [7] suggests that drugs that modulate these types of cellular regulations may be promising candidates for CAVD treatment [4].

The cell-driven or cell-affecting processes related to CAVD include lipid infiltration, inflammation, the endothelial-to-mesenchymal transition, fibrosis and osteogenesis [1, 8]. Of particular interest to the present study was the finding that an osteoblast-like lineage may originate from valvular interstitial cells (VIC) [4, 8, 9]. This VIC population is responsible for the maintenance of the extracellular matrix (ECM] in the valve cusp tissue and consists of various subpopulations, including osteoblastic VICs [10]. Assuming that osteoblastic VICs are indeed responsible for the processes of neo-osteogenesis, differentiation of VIC preparations into the osteoblastic status should be possible *in vitro*. Media formulations that are used for differentiation of mesenchymal stem cells, such as those containing ascorbic acid phosphate, dexamethasone, and β-glycerophosphate, have been tested previously [11]. Ascorbic acid induces the secretion of type I collagen and β-glycerophosphate is the source for phosphate in hydroxylapatite, whereas dexamethasone induces Runx2 expression (e.g. by β-catenin–mediated transcriptional activation) [12].

The signalling pathways involved in AV osteogenic processes are Notch, bone morphogenetic protein and Wnt/β-catenin signalling [9]. The Wnt/β-catenin pathway, in particular, is considered relevant for osteoblastic differentiation for CAVD at the molecular level [13–16], as western blotting and RT-PCR analyses have confirmed the upregulation of β-catenin in calcified aortic valves and in bone in the stenotic aortic valve [17]. Oxidized low density lipoprotein leads to AV calcification *in vitro* and *in vivo* and LRP5/Wnt signal and β-catenin are related with cardiovascular calcification [14, 15, 17–19]. The myofibroblastic differentiation processes of VICs have also been related to the Wnt/β-catenin signalling that depends on matrix stiffness and TGF-β1 [20]. Stimulation of porcine VICs by oxidised low density lipoprotein also induces β-catenin expression, leading to the conclusion that Wnt/β-catenin signalling plays a key role in osteoblastic VIC differentiation, thereby contributing to CAVD [14]. Similar results were published by Gao et al., who demonstrated an involvement of the Wnt/β-catenin signalling pathway in osteoblastic VIC differentiation monitored by western blotting and/or RT-PCR detection of extracellular matrix proteins and respective gene markers [21]. Porcine VICs also showed upregulation of Wnt3a and β-catenin expression in response to treatment with angiotensin II, leading to the assumption that angiotensin II also induces AV calcification [22].

Fang et al. linked the Wnt/β-catenin pathway to Sox9, a master regulator of chondrogenic lineage, and revealed that β-catenin limits the expression and nuclear localisation of Sox9 in cultured chicken aortic valves and in adult mice aortic valve nodules [13, 16, 23]. Sox9 binds and represses transactivation of the osteogenic glycoprotein Spp1, and a reduced Sox9 function has been defined as a basis for calcific valvular disease [24, 25]. Activation of Wnt signalling induces osteoblast differentiation but suppresses chondrocyte differentiation of mesenchymal stem cells, and Sox9 expression is increased after genetic inactivation of β-catenin [26, 27]. Reduction in Wnt/β-catenin signalling by treatment of cultured chicken aortic valves with the tankyrase inhibitor XAV-939 resulted in higher expression and nuclear localisation of Sox9 [16].

XAV-939 inhibits the Wnt/β-catenin signalling pathway by stabilising Axin [28], a scaffolding protein. Axin is one concentration-limiting factor for β-catenin degradation as it anchors proteins involved in the degradation complex [28, 29]. The stabilisation of Axin therefore reduces the Sox9 activity induced by β-catenin signalling and the formation of chondrogenic ECM proteins—and thereby, chondrogenesis—resulting in a disorder resembling myxomatous valve disease [16]. Indeed, the dysregulation of Wnt/β-catenin signalling has also been correlated with progressive myxomatous valve disease [30]. The observation of an increased Sox9 mRNA expression in the degenerative mitral valve and calcified aortic valve, with the highest amounts in the cartilage phenotype of diseased mitral valves, has led to the assumption that bone differentiation in degenerative valve lesions results, on the one hand, in a cartilage phenotype in mitral valves and, on the other hand, in a bone phenotype in aortic valves [17]. This assumption is supported by histological investigations showing downregulation of Sox9 in human calcified AVs near the regions of calcification and localisation of the remaining Sox9 in these areas in the cytoplasm as an inactive form [31, 32].

In addition to Wnt/β-catenin signalling, the Notch pathway has been implicated in regulation of Sox9, although Sox9 does not seem to be a direct Notch target [33]. Notch and Sox9 were downregulated in porcine VICs in spontaneously calcifying cultures, whereas expression of osteogenic markers was increased, and the loss of Notch signalling has been postulated to contribute to aortic valve calcification via a Sox9-dependent mechanism [33]. Co-cultured endothelial cells have the ability to protect VICs from calcification by sustaining the Sox9 nuclear localisation [31]. Therefore, a drug-based intervention that can stabilise Sox9 phosphorylation and nuclear localisation is suggested [31].

The aim of the present project was to analyse the potential anti-calcific effect of XAV-939 and the expression of Axin, β-catenin and Sox9 in human VIC cultures in terms of potential regulatory mechanisms for osteogenic differentiation via Wnt/β-catenin signalling versus Sox9 driven chondrogenesis. Calcification was induced *in vitro* in human VIC cultures by incubation with a combination of ascorbic acid phosphate, dexamethasone and β-glycerophosphate and the mineral deposits were characterised.

## Materials and methods

### Patient materials

Nineteen aortic valves were used to isolate VICs for cell culture after the written informed consent form was signed by patients (no minor patients). The study was approved by the ethics committee of the Dresden University (*Ethikkomission an der TU Dresden*, *registration number EK429102015*). The valves were replaced with prostheses in the daily routine of cardiac surgery. Thirteen patients were male and six were female (average age 65.2 ± 11.8 years). Three of the 19 valves prepared for the present study were bicuspid and 16 were tricuspid.

### Cell culture

Human VICs were isolated as described previously [34]. After enzymatic removal of the endothelial cell layer with a mixture of collagenase (Serva, Collagenase NB8 Broad Range 0.3 PZ U/ml) and dispase II (Sigma; 0.81 U/mg), the cusps were minced with scalpels and further digested with collagenase. The released cells were filtered, the collagenase was inactivated and the cells were collected by centrifugation, and counted. The resulting VIC populations were plated on collagen type 1 (BD) coated culture plastic and cultured in DMEM (10% foetal calf serum supplementation) in a humidified atmosphere at 8% CO_2_ until used for *in vitro* induction of calcification. No CD31 positive cells were detected in three independent cell preparations analysed by immunofluorescence staining, and 83.2 ± 8.3% of the cells strongly expressed αSMA (not shown). Calcification was induced by seeding 2000 human VICs/cm^2^ (passages 1–6) in 6-well plates (not collagen coated). After 72 hours, the culture medium was replaced with ADG medium (ADGM; 50 μM **A**scorbic acid phosphate, 10 nM **D**examethasone, 10 mM β-**G**lycerophosphate). ADGM and ADGM including XAV-939 were freshly prepared weekly and replaced every 2–3 days until mineral precipitations were visible (the maximum incubation time was 35 days without passaging). Control cells were co-cultured in DMEM. As soon as mineralisation was visible in the ADGM-culture, the experiment was stopped and the end points were analysed. XAV-939 was solubilised in DMSO and freshly diluted to a final concentration of 1 μM in culture medium. Due to the high dilution factor (1:6000), DMSO was not added to the DMEM control wells. Two plates were prepared in parallel for calcium quantification and to isolate protein extracts using RIPA buffer. Impact of XAV-939 inhibitor treatment on cell viability of human VICs was investigated by MTT-assay (CellTiter-Blue Cell Viability Assay, Promega) after 72h treatment at concentrations of 10, 1 and 0.1 μM revealing (n = 4). To perform MTT-assay cells were seeded at a density of 2000 cells per well of a 96-well-plate. After 72h XAV-939 containing media were added and replaced after additional 24h. MTT-assay was performed after 72h of inhibitor incubation according to the manufacturer’s instructions in a 96-well format.

### Histological staining

The calcified cell layer in histological setups was analysed by washing the 6-well plates containing the ADGM induced cell monolayers and the DMEM controls twice with DPBS (Dulbecco's phosphate-buffered saline), adding 400 μl DPBS and scraping and separating the cell layers from the wells. The separated cell material was transferred to a reaction tube and centrifuged at 200 g for 10 min to precipitate the material. The pellet was fixed in 4% buffered formalin solution. Samples were paraffin embedded and sliced. Movat Pentachrom staining was performed according to the manufacturer’s (Morphisto) instructions. Calcification was visualised by staining the slices with von Kossa and Alizarin red stain according to standard methods. As a control, sliced specimens were decalcified after rehydration by overnight incubation in Osteosoft solution. Images were acquired using a Slide Scanner (Axio ScanZ.1 by ZEISS) or Zeiss Observer Z.1.

### Infrared spectroscopy

One induced cell culture preparation was used for initial investigation of the precipitates via Fourier Transformed Infrared (FT-IR) spectroscopy. The cell culture well was washed twice with DPBS and the cells were carefully scraped from culture plastics and centrifuged at 300 g. The resulting pellet was directly transferred to embedding media and frozen at -80°C. Cryosections with a thickness of 20 μm were transferred to a CaF_2_ object slide. Sections of cell culture pellet sample were analysed and compared to a formaldehyde-fixed, calcified human AV sample.

IR spectroscopy images were collected in transmission mode using a Vertex 70 FT-IR spectrometer coupled with a Hyperion 3000 infrared microscope (both from Bruker Optik GmbH, Ettlingen, Germany). The imaging detector was a Santa Barbara focal plane MCT 64 x 64 array detector. The 15× Cassegrainian objective with a numerical aperture of 0.4 imaged a sample area of approx. 175 × 175 μm^2^. The compositions of individual infrared images were captured, subject to the size of the area investigated. Background spectroscopy images were recorded from the pure CaF_2_ object slide before the tissue sections were investigated. For all measurements, 100 interferograms (scans) were co-added. The interferograms were Fourier transformed applying Happ-Genzel apodisation and a zero filling factor of 1. Spectra at a resolution of 6 cm^-1^ of the sample image were rationed against the spectra of the background image and transformed into absorbance values.

The spectral data were evaluated using the Matlab Package (Version 7, Math Works Inc. Natick, MA, USA). Only the so-called fingerprint region between 950 and 1800 cm^-1^ was considered for data volume minimisation. Data pre-processing involves a removal of outliers, a baseline correction and a normalisation of each absorbance value of a spectrum to the integral absorbance. Outliers are spectra that are obviously not associated with tissue or spectra with a maximum absorbance value larger than 1.5. The baseline of each spectrum was corrected using the msbackadj function of the Statistics Toolbox of Matlab. Afterwards, the spectra were area-normalised to eradicate spectral differences due to sample thickness or variation in the density of cellulose fibres. K-means cluster analysis of with four clusters was performed with the k-means function of the Statistics Toolbox.

### Protein isolation and quantification

Whole cell protein was isolated from the wells after removal of the medium and washing the culture twice with DPBS. A 200 μl volume of cold (4°C) RIPA buffer containing protease inhibitors and phosphatase inhibitors was added to each well and incubated for 10 min on ice. Cellular material was scraped, transferred to tubes, sonicated to disrupt the cells and pelleted by centrifugation. The protein content of the supernatant was determined in a 96-well format using a Pierce BCA Protein Assay Kit (Thermo Fisher Scientific) according to the manufacturer’s instructions.

### PAGE and western blotting

Proteins were separated by 10% SDS-PAGE and transferred onto nitrocellulose membranes (Roth). The membranes were blocked overnight at 4°C or for 1.5 h at room temperature (RT) in TBS or PBS, respectively, containing 0.1% Tween20 and 5% milk powder. After three washes with buffers supplemented with 0.1% Tween20, the membranes were exposed to the following antibodies in their respective dilutions: Anti-Alkaline Phosphatase (abcam, Ab54778, 1:200, TBS), Anti-Axin1 (R&D Systems, AF3287, 1:400, TBS), Anti-β-catenin (BD, 610153, 1:1000, TBS), Anti-Non-Phospho (Ser33/37/Thr41) β-catenin (Cell Signaling, #4270, 1:1000, PBS), Anti-Sox9 (abcam, Ab185230, 1:1000, TBS) and Anti-GAPDH (control, Antibodies-online, ABIN1107320, 1:2000, TBS). Primary antibodies were incubated for 1 h at RT or overnight at 4°C. Membranes were washed, incubated with secondary anti-mouse (Cell Signalling, 7076P2, 1:3000), anti-rabbit (Cell Signalling, 7074S, 1:3000) or anti-goat (R&D Systems, HAF109, 1:1000) immunoglobulin/HRP (1 h, RT) and treated with western blotting luminol reagent (Merck) for 1 min. The luminol signal was detected using a PHASE-Detection camera (PHASE) at defined time points. Semiquantitative analyses of expression levels relative to GAPDH were performed with Image Studio Lite software (https://www.licor.com/bio/products/software/image_studio_lite), with expression in DMEM control wells set as 100%.

### Immunofluorescence staining

Human VIC cultures were seeded on glass slides at a density of 3 × 10^4^ cells per slide. ADGM, DMEM-XAV-939 and ADGM-XAV-939 were added after 72 h in conventional culture. After 14 days of incubation in the different media, the slides were washed twice with DPBS and fixed in 4°C acetone for 10 minutes. Cells were permeabilised with PBS containing 0.2% TritonX100 for 10 minutes and blocked in PBS containing 1%BSA for 30 minutes. Sox9 antibody was diluted 1:1000 in PBS/BSA, added to the cells and incubated for 1 h at RT. As a control, an isotype monoclonal rabbit antibody IgG (abcam, Ab125938) was used at an equivalent concentration. After washing of the slides, a secondary donkey Anti-Rabbit IgG 488 (1:500, abcam, Ab150073) antibody in PBS/BSA was incubated for 1 h at RT. The cells were DAPI stained and washed with PBS twice and then embedded in fluorescent mounting medium (Fluoromount G, Invitrogen). Staining was evaluated and images were acquired using a Zeiss Observer Z.1 Apotome fluorescence microscope. Fluorescent nuclei were counted in relation to DAPI-stained nuclei and the percentages of Sox9-positive nuclei were evaluated.

### Calcium quantification

The calcium content in the induced cell culture wells was determined by replacing the medium, washing the dish twice with calcium free DPBS and adding 1 ml 0.1 M nitric acid. The sample was incubated at RT overnight and calcium ions were quantified using a Spektroquant Calcium-Test Kit (Merck KGaA, measuring range 0.2–4.0 mg/l [5–100 μmol/l] Ca, SD of the method ± 0.032 mg/l [0.8 μmol/l] Ca), according to the manufacturer’s instructions and after relevant dilutions.

The calcium content was related to total protein by removing the nitric acid solution, washing the wells twice with DPBS, adding 500 μl of a 0.05 M NaOH/0.1% SDS solution per well and incubating overnight at RT. Protein concentration was determined as described above. For better comparability, the results were calculated in units of mol/(kg protein).

### Statistical analysis

The Wilcoxon matched pairs test was used to test statistical significance of calcium content of DMEM vs ADGM cell culture samples.

A two tailed paired t-test was used to test alkaline phosphatase expression levels in the ADGM vs DMEM conditions, and a Wilcoxon matched pairs test was used to compare DMEM-XAV-939 vs ADGM-XAV-939. The statistical significance of SOX9 nuclear localisation was tested with a paired t-test.

One-way-ANOVA and the Newman Keuls multiple comparison test were applied to Axin1, β-catenin and SOX9 western blot data and to quantify the calcium content in the experiments with the four arms: DMEM, ADGM, DMEM-XAV-939 and ADGM-XAV-939.

Analyses were performed using Graph Pad Prism 5 software (GraphPad Software, Inc.). Data are presented as mean ± SD. Significant differences are indicated by asterisks.

## Results

### Induction of calcification

**Fig 1:**
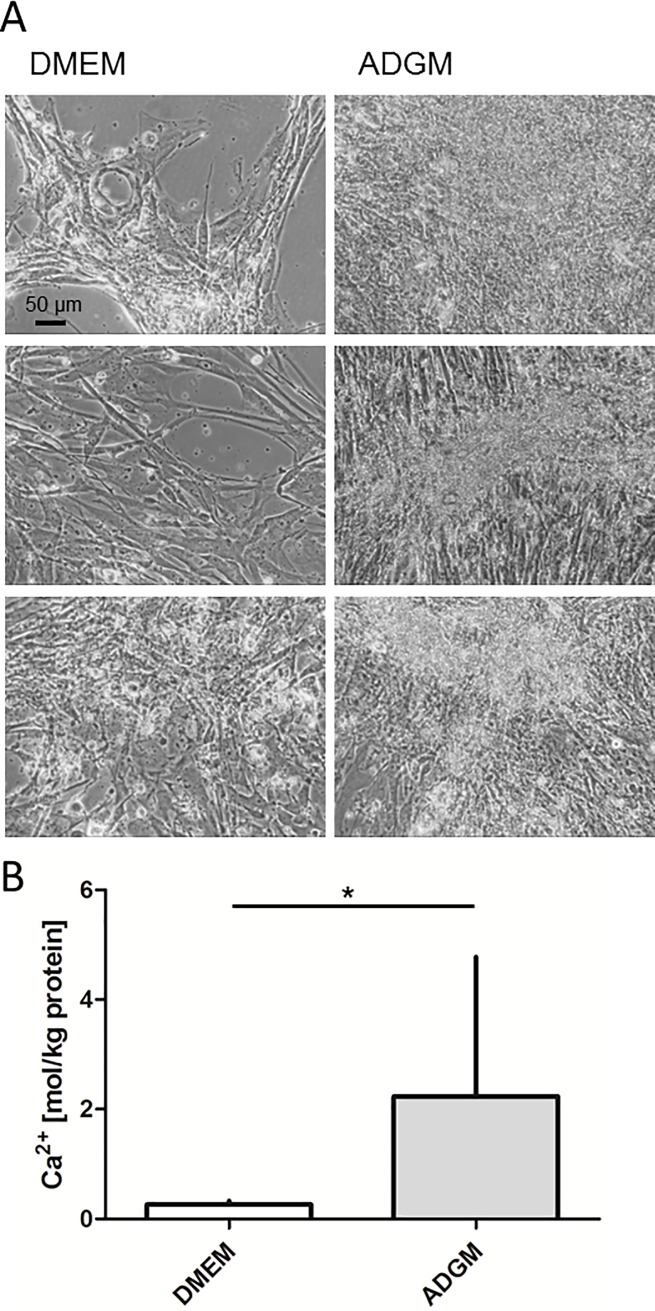
Induction of Calcification in VIC cultures *in vitro*. A) Microscopic visualisation of standard VIC cultures in DMEM, showing a light to brownish calcification precipitate after induction in ADGM. Three different human VIC preparations are shown. B) Quantification of calcium ions in control (DMEM) vs ADGM-induced cultures normalised to total protein content reveals a significant increase (n = 11).

Nineteen individual human VIC cultures were used to study calcification processes induced by culture in ADGM. The ADGM-induced cultures showed a light to brownish precipitate after different culture times from 14 to 33 days (Fig 1A, three individual examples). No calcification was observed in the DMEM culture. In 11 (58%) of the 19 analysed preparations, an enrichment of calcium was evident. The calcium content was calculated in relation to the protein content. Individual VIC cultures that showed significantly higher calcium contents in ADGM wells in comparison to DMEM were defined as induced and were implemented in subsequent substance testing experiments. Calculation of the results of all eleven induced VIC cultures analysed herein revealed a significant enrichment from 0.3 ± 0.2 mol/kg protein in the DMEM controls to 2.2 ± 2.5 mol/kg protein (p<0.05; Fig 1B) in the ADGM-induced cells. The ADGM cultures showed a high biological variability that is reflected in the calcium content and in the time needed for induction.

Two of the inducible VIC preparations were isolated from valves from female patients and nine from male individuals. The cell preparations that did not calcify resulted from aortic valve tissue from four female and four male patients. Nine and seven of the aortic valves the inducible vs. non-inducible VIC preparations resulted from were tricuspid, respectively. There was no statistically significant relationship of these patient parameters to the calcification potential.

**Fig 2:**
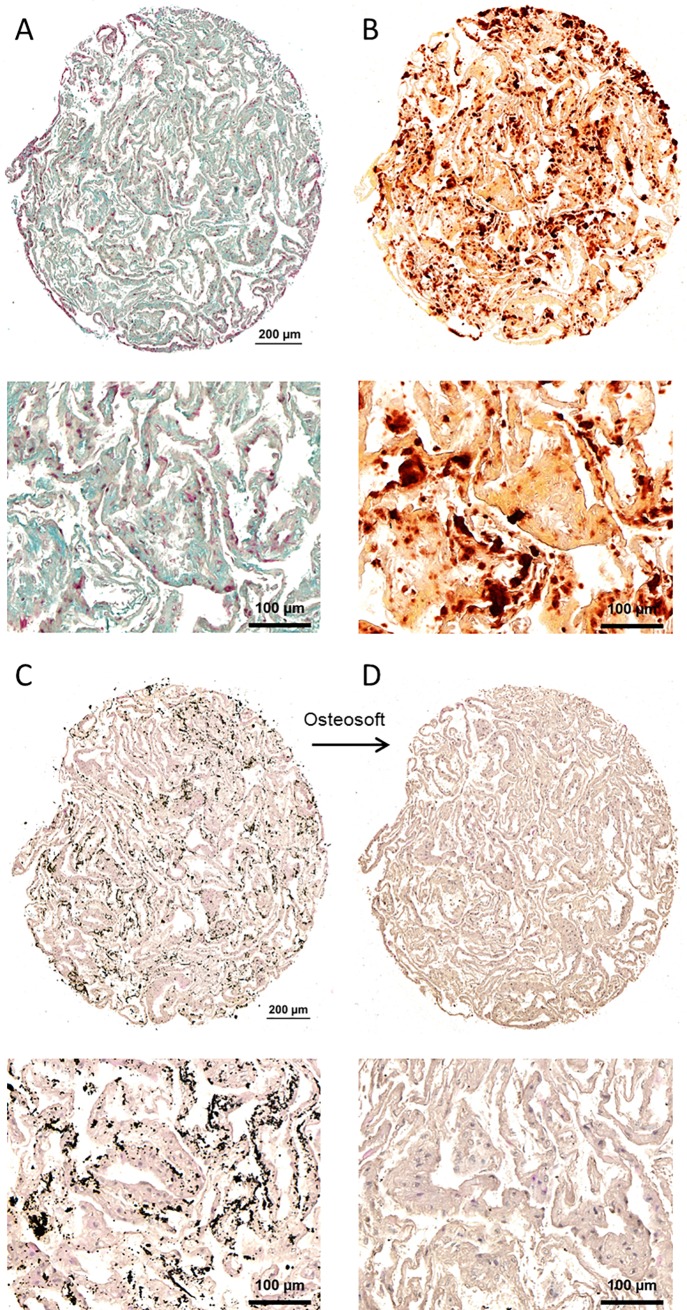
**Histological analysis of VIC culture pellet after ADGM induction.** (lower picture represents a higher magnification of a portion of the complete pellet specimen) A) Homogenous extracellular matrix distribution visualised by Movat Pentachrom stain. Cell nuclei are coloured dark red. B) Alizarin red staining confirmed calcification of *in vitro* cell culture samples.C) Von Kossa positive calcification signals in the induced VIC cell culture pellet are eliminated by treatment of the histological section with Osteosoft solution. The sections were HE stained; cell nuclei are coloured blue.

Histological analysis of ADGM-induced VIC cultures, when compared to a homogeneous ECM (Fig 2A, Movat Pentachrom stain), showed calcification that gave intense Alizarin red and von Kossa staining (Fig 2B & 2C). Incubation of sections overnight with Osteosoft-solution (for decalcification) eliminated the von Kossa signal (Fig 2D). Respective control stainings of a DMEM-cultured VIC pellet are shown in S2 Fig and revealed no calcific mineral.

**Fig 3:**
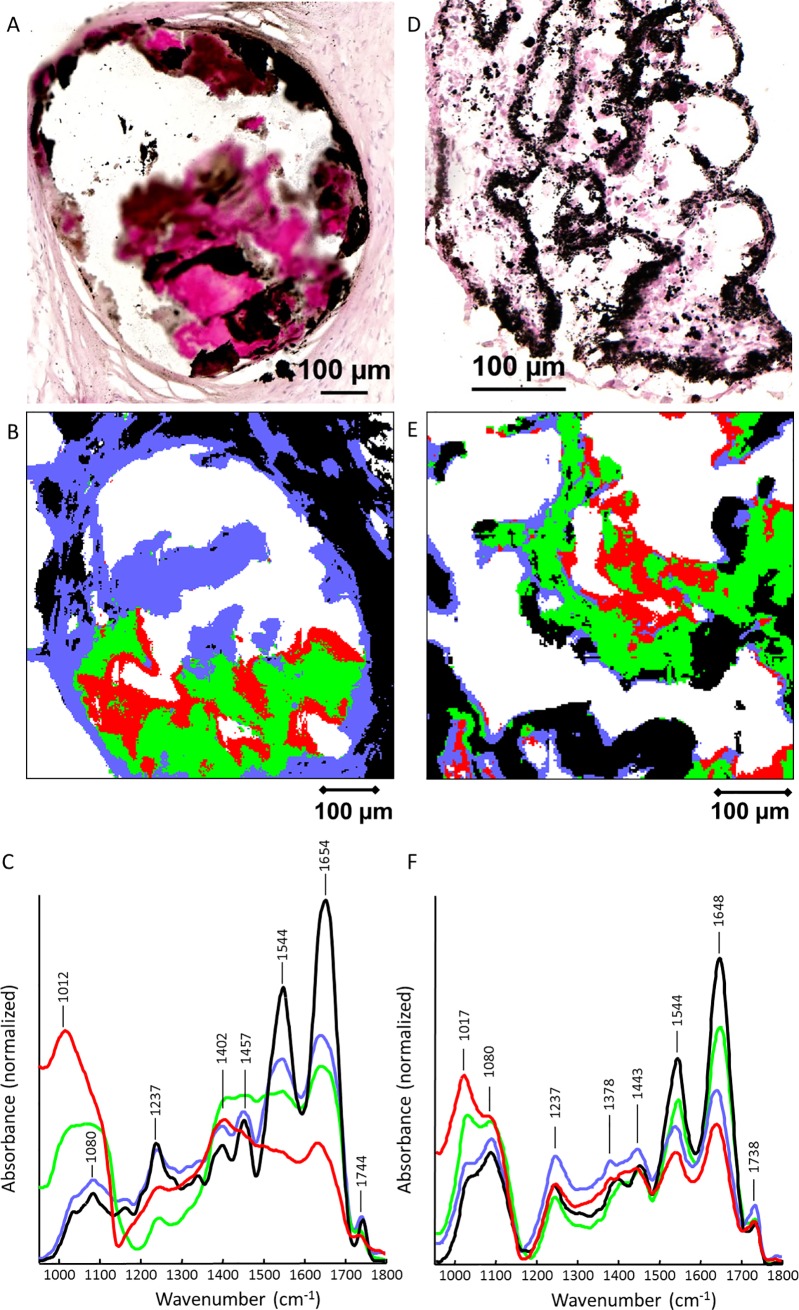
**IR spectroscopy—Results of infrared spectra k-means cluster analysis.** A) von Kossa staining of a human aortic valve section reveals positive calcification signals in the mineralised areas; B) Map of k-means cluster analysis; C) Corresponding centroid spectra. White pixels indicate areas without tissue and were removed from the data set. D) to F) Von Kossa staining and corresponding IR analyses of pelleted calcified cell cultures compared to A) to C). Both samples exhibit black von Kossa staining signals and similarities in their centroid spectra.

A calcified VIC cell culture was prepared for IR-spectroscopy analysis by scraping the material, pelleting the cells by centrifugation and cryo-sectioning. A patient’s AV was analysed in parallel for comparison of the spectra. Fig 3A shows a histological section of this human AV and Fig 3B shows the cluster analysis results for the spectral data set of a parallel section. The corresponding centroid spectra are presented in Fig 3C. The cluster map reveals two different areas: the tissue is mainly represented by blue and black pixels, whereas the calcific phase is indicated by green and red pixels. The centroid spectrum of the blue and black pixels shows the typical spectral features of tissue with strong amide I, II and III bands at 1654, 1544 cm^-1^ and 1237 cm^-1^, respectively, and increased carbonyl stretching mode of phospholipid esters at 1744 cm^-1^ [35–37]. Other bands located at 1402 and 1457 cm^-1^ were assigned to CH_x_ groups of lipids [35]. Spectra visualised by red and green pixels were dominated by a strong signal of hydroxylapatite phosphate groups between 1000 and 1150 cm^-1^ [35, 36] with less intense bands resulting from the tissue of the specimen (amide I, II and III and phospholipid esters).

Histological analysis after von Kossa staining of the cell pellet revealed a positive calcification signal. Respective cluster analysis of this sample is shown in Fig 3E, and refers back to Fig 3B. Centroid spectra (Fig 3F) exhibit a similar pattern like spectra shown in Fig 3C. The calcific phase is represented by red clusters, while black pixels indicate noncalcified tissue. The centroid spectra of blue and green clusters show both proteins and mineralisation, so these areas are interpreted as a conglomerate of cellular organic material and mineralisation. Therefore, the calcification of the cell culture pellet sample shown via von Kossa staining was verified as calcium hydroxylapatite by the mineral IR-spectroscopy data.

### Impact of XAV-939 treatment on *in vitro* VIC calcification

**Fig 4:**
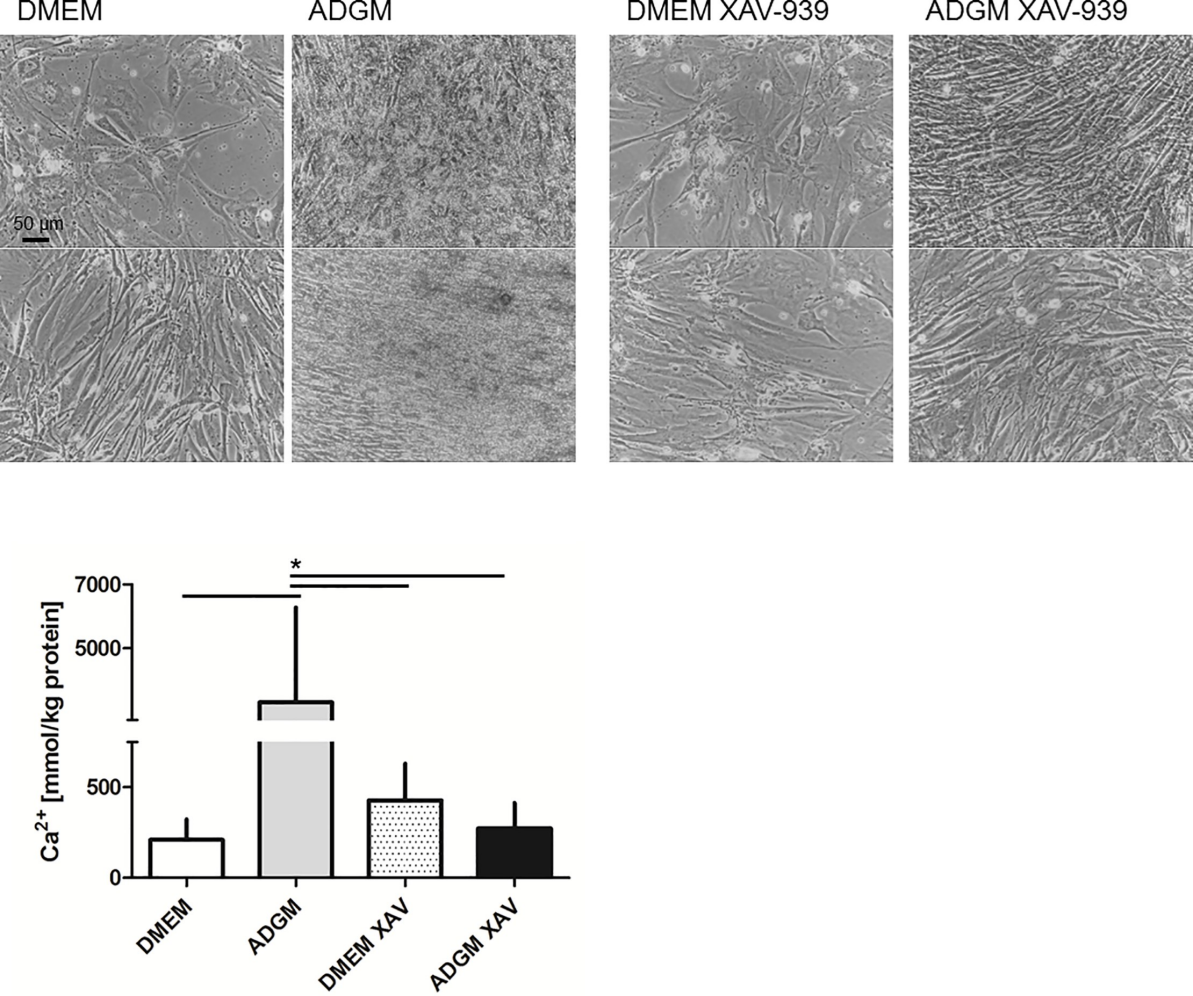
Additional treatment of VIC cultures with XAV 939 prevents *in vitro* calcification during ADGM induction. A) No mineral precipitate is visible if VIC cultures are co-incubated with XAV 939 in ADGM, shown here for two examples of human VIC preparations. B) XAV 939 treatment reduces the calcium concentration in ADGM-induced cultures to levels comparable to control DMEM cultures. The calcium levels were significantly higher in the ADGM-induced cultures than in the other conditions (n = 7).

**Fig 5:**
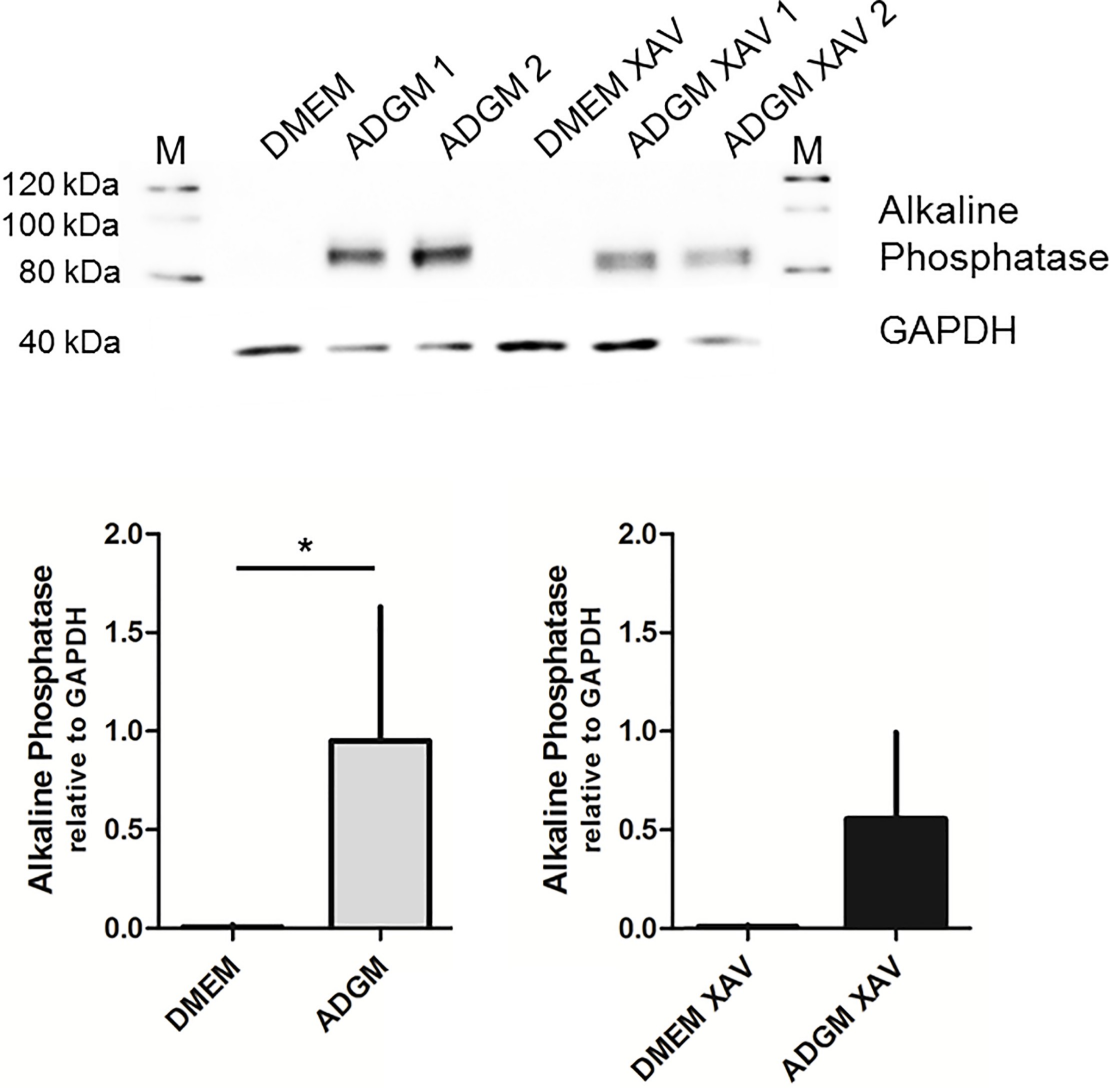
Expression of alkaline phosphatase in ADGM–and XAV-939–treated VICs. Alkaline phosphatase is significantly upregulated in ADGM-induced cell cultures compared to the DMEM control. This induction was not statistically significant when DMEM and ADGM cultures were additionally treated with XAV-939 (n = 5).

A negative effect of XAV-939 treatment (10, 1, 0.1 μM) on MTT-based viability of VICs was not detected (S1 Fig). The impact of XAV-939 treatment on the induction of calcification in VIC cultures was investigated using seven individual VIC preparations. *In vitro* calcification was induced from 0.2 ± 0.1 mol/kg protein in the DMEM controls to 3.3 ± 3.0 mol/kg protein (p<0.05) after culturing in ADGM. When XAV-939 was added to the ADGM culture wells, no calcification was apparent (Fig 4A) and the calcium content was 0.3 ± 0.1 mol/kg protein, or comparable to DMEM control and DMEM-XAV-939 levels (0.4 ± 0.2 mol/kg protein; Fig 4B). Therefore, the XAV-939 treatment significantly inhibited the calcification process in VIC cultures. Expression of alkaline phosphatase was significantly upregulated in ADGM-induced VIC cultures, whereas almost no alkaline phosphatase expression was detected in DMEM cultures. No significant ADGM-induced upregulation was noted when cells were incubated in ADGM-XAV-939 when compared to the DMEM-XAV-939 control (Fig 5).

### Expression of Axin1, β-catenin and Sox9

Upregulation of β-catenin and Sox9 expression is involved in osteoblast and/or chondrocyte differentiation. Inhibition of tankyrase by XAV-939 stabilises Axin1, which is one member of the β-catenin destruction complex. The expression levels of Axin1, β-catenin and Sox9 were analysed by western blotting of protein lysates of VIC standard cultures and compared to ADGM-induced calcified cells and cells co-incubated with XAV-939.

**Fig 6:**
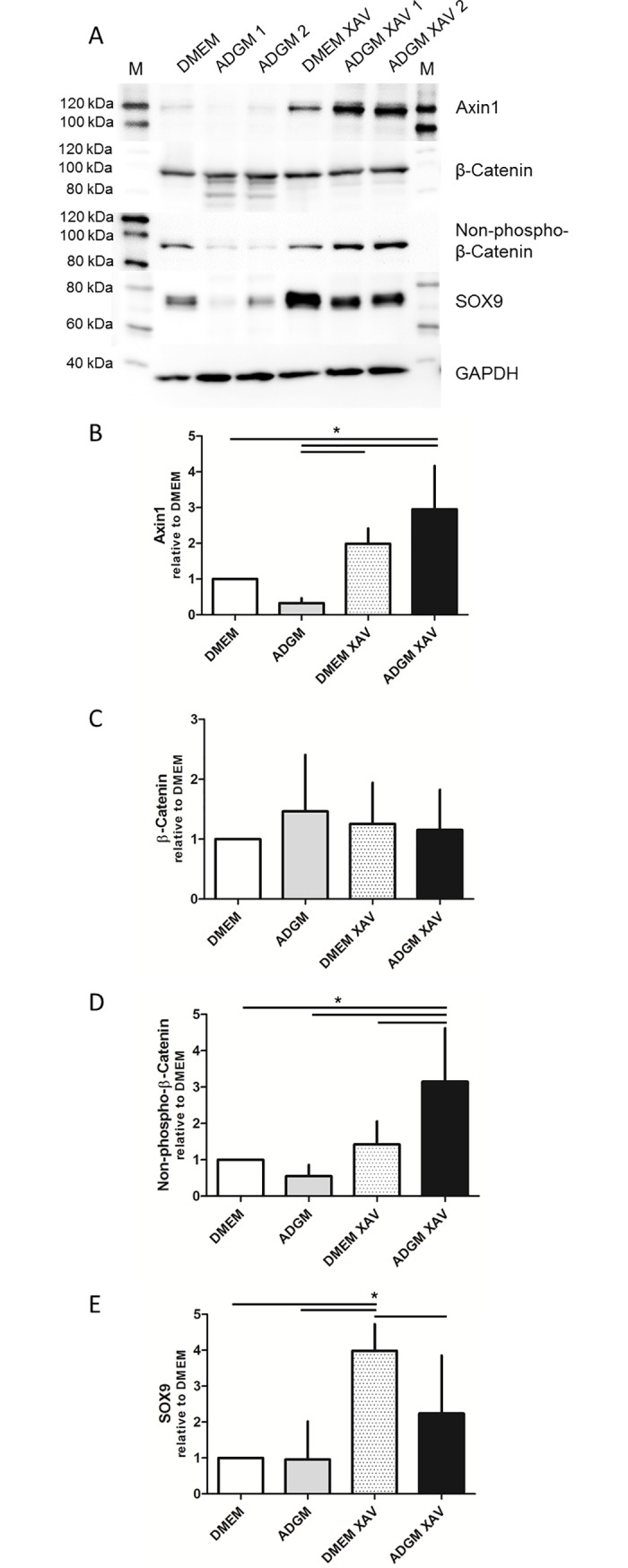
Western blot analyses of ADGM-induced and XAV-939–treated cultures. A) Western blot analysis of the Wnt signalling proteins Axin1, β-catenin (total and non-phospho-protein) and Sox9. B) Axin1 is significantly upregulated *in vitro* in XAV-939–treated VICs in DMEM and ADGM (n = 3). C) Total β-catenin expression does not differ in the media conditions and does not depend on XAV-939 treatment (n = 5). D) Non phospho β-catenin is upregulated in XAV-939–treated conditions (significant upregulation in ADGM with XAV 939) and downregulated in ADGM induced VICs (n = 5). E) Sox9 is significantly upregulated in VIC cells co-incubated in DMEM with XAV-939, whereas this expression is reduced in VIC cells co-incubated in ADGM with XAV-939 (n = 4).

As expected, Axin1 was stabilised in VIC cultures after co-incubation with XAV-939 (Fig 6A & 6B). A significantly higher Axin1 signal of 294.7 ± 121.5% was detected in VICs treated with ADGM-XAV-939 when compared to the DMEM control (p<0.05; Fig 6A). Cultures treated with DMEM-XAV-939 showed a nearly 2-fold upregulation of Axin1 expression (198.4 ± 43.2%). ADGM treatment alone did not induce Axin1 expression. The results did not support the hypothesis of overexpression of β-catenin in calcification-induced VICs, as the total protein expression did not differ significantly (Fig 6A & 6C). In addition, expression of the non-phospho-β-catenin (the active form), at 54.9 ± 0.3%, was significantly lower in cultures that exhibited calcification precipitates when compared to ADGM-induced cultures that showed high expression levels after the addition of XAV-939 (314.6 ± 147.9%; p<0.001), even though XAV-939 should promote β-catenin phosphorylation and degradation by stabilising Axin (Fig 6A–6D). Sox9 expression was significantly higher, at 398.4 ± 74.0% (DMEM-XAV-939; p<0.001) and 224.2 ± 161.3% (ADGM-XAV-939), in cultures treated with XAV-939 (Fig 6A & 6E). The DMEM-XAV-939 condition showed the highest expression levels and was, in turn, significantly (p<0.05) reduced in the ADGM-XAV-939 condition, and it exhibited the lowest expression in the arms without XAV-939 treatment.

### Immunofluorescence staining of Sox9

**Fig 7:**
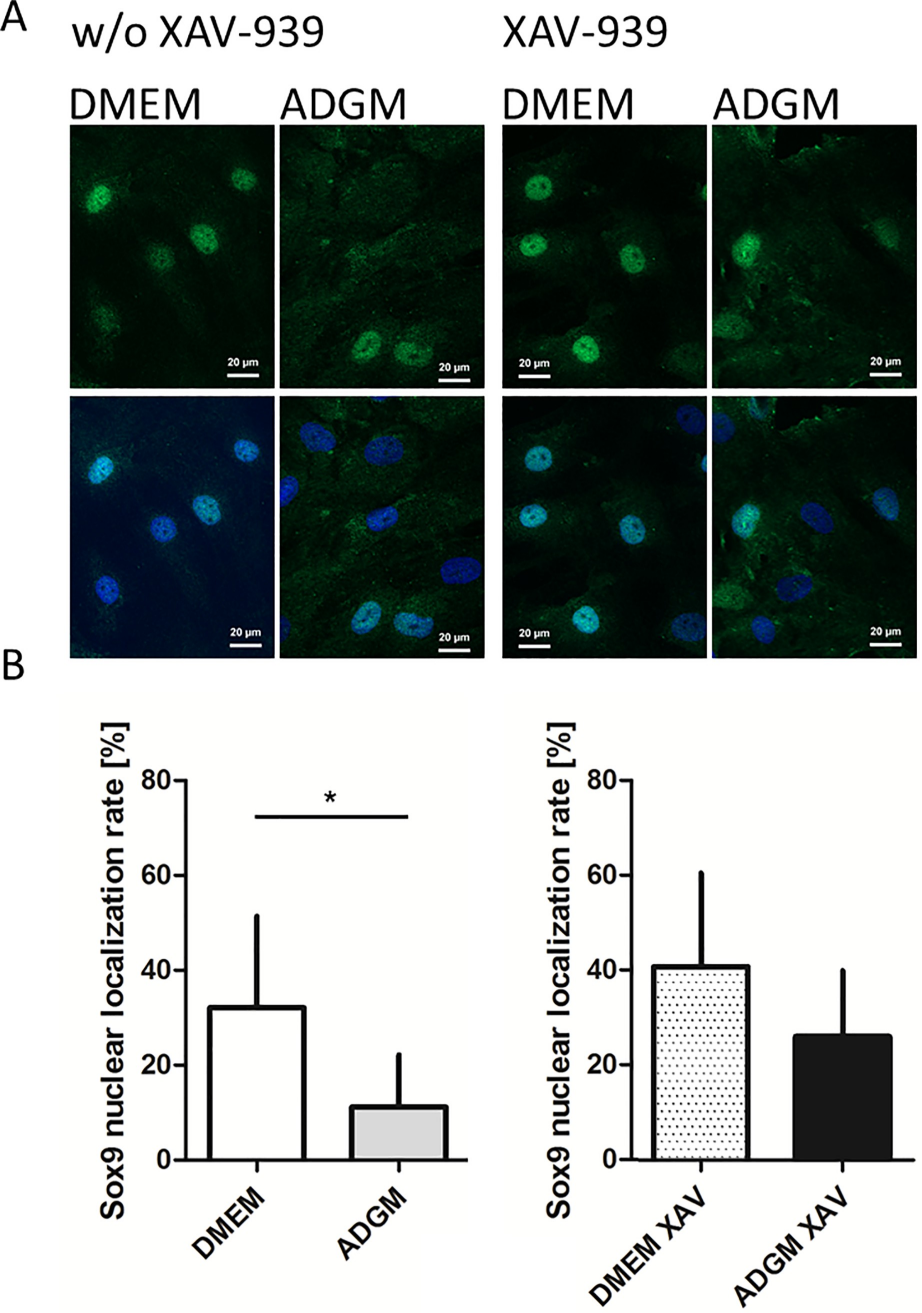
**Nuclear localisation of Sox9 in human VICs in different cell culture formulations.** A) Example showing the nuclear localisation of Sox9 in a human VIC culture incubated in DMEM, ADGM and the respective XAV-939-treated conditions. B) Rate of Sox9 nuclear localisation in VIC cultures is significantly lower in the ADGM condition than in the DMEM control. This increase was not statistically significant after additional treatment with XAV-939 for both conditions (n = 5; only inducible cultures were investigated).

Human VICs cultured on glass slides in DMEM, ADGM, DMEM-XAV-939 and ADGM-XAV-939 were stained for Sox9 expression in an immunofluorescence setup. Evaluation of Sox9 nuclear localisation revealed a rate of 32.1 ± 19.3% in VICs cultured in DMEM and a significant reduction to 11.2 ± 11.1% in the ADGM condition (Fig 7A & 7B). Incubation of VIC cells in DMEM-XAV-939 control gave a rate of 40.7 ± 8.8%, but this difference was not statistically significant when compared with the ADGM-XAV-939 condition (26.6 ± 6.2%; Fig 7B).

## Discussion

The tankyrase inhibitor XAV-939 is able to prevent *in vitro* induction of calcification in human VIC culture preparations. As expected, XAV-939 treatment resulted in the stabilisation of Axin, a member of β-catenin degradation complex [28]; therefore, XAV-939 functioned as an inhibitor of the Wnt/β-catenin signalling pathway. This reduced β-catenin signalling, in turn, was expected to lead to the induction of Sox9 [16]; however, the XAV-939–treated samples showed a lower expression of the active form of β-catenin in the calcifying VICs, indicating that β-catenin activity is not the relevant biological process in this cell system. Instead, β-catenin signalling is active in the DMEM control and is induced after treatment with XAV-939 in the DMEM and even more strongly in the ADGM condition. The phosphorylation status of Axin is important for functionality, and one explanation for the lack of an effect on the non-phospho-β-catenin level may be a different post-translational modification (e.g. the phosphorylation of the protein [29]), but this needs further investigation in future studies. The expression of Axin1 is not elevated in the ADGM condition.

The Sox9 protein expression is upregulated when cultures are incubated with XAV-939 in the DMEM and ADGM conditions, but this Sox9 protein upregulation does not correlate with a reduced expression of the non-phospho-β-catenin. The induction of Sox9 expression in the DMEM-XAV-939 condition vs the DMEM control, where non-phospho-β-catenin is expressed and where Wnt signalling seems to be active, is of particular interest. The total protein level indicates a significant reduction in the rate of Sox9 nuclear localisation detected by immunofluorescence in the ADGM-induced calcified samples. The addition of XAV-939 to the ADGM samples abrogated this reduction when compared to the DMEM-XAV-939 control. As expected, the higher SOX9 activity resulted in no induction of calcification. The relationship to Wnt/β-catenin signalling in this context and the phosphorylation status of Sox9 needs further investigation, as the phosphorylated protein form is active and localised in the cell nucleus [38].

XAV-939 is one member of a large set of small-molecule inhibitors of the Wnt/β-catenin pathway [28, 39, 40]. Targeting of this pathway in clinical or experimental settings and the applicability of the inhibitors have several limitations, so ongoing drug design is focused on identifying additional specific inhibitors that have fewer side effects [39, 41–43]. Members and proteins of the Wnt/β-catenin signalling or Notch signalling pathways may be new targets for drug development to prevent the progression of AV stenosis, since these signalling pathways are believed to be involved in osteogenesis in human AV disease [4, 9, 17, 33, 44]. Apart from the aim to find clinically relevant substances, drug design and innovation focusing on small inhibitor molecules can be tested in *in vitro* settings in basic research studies to define mechanistically important processes.

The model system analysed in the present study was *in vitro* cultured human VICs showing induced calcification with ADGM. Only 58% of the VIC preparations showed an induction of calcification during an incubation time of up to 35 days. Cell preparations are heterogeneous, consisting of various subpopulations, so the *in vitro* calcification processes are expected to differ. The rate of calcification induction can depend on individual culture preparations and their responsiveness to ADGM, as well as the time in culture. In addition aspects like gender of patients the VICs originate from and bi-vs. tricuspidal valve development have to be considered in studies investigating a larger sample set.

Calcified AV exhibit an increased prevalence of cells positive for smooth muscle cell (SMC) markers [45]. SMCs of vascular origin (VSMCs) are able to calcify *in vitro* [46–48] and are responsible for vascular calcification [47]. Other vascular osteo-progenitors originate from immature circulating bone marrow subpopulations [49]. Determining the frequency and origin of SMCs and bone marrow subpopulations in human aortic valves and the contribution of these cells to *in vitro* calcification processes will require further culture experiments.

Limitations of the present study include the use of human VICs isolated from diseased human AV tissue and the heterogeneity of the isolated cell population. However, the use of human VICs instead of cells of porcine, bovine or ovine origin isolated from very young individuals may be advantageous, as most of the mechanistic studies on VICs have been performed on cellular material from other species [11, 16, 50, 51]. Indeed, the use of human AV tissue cell preparations in the present study may be one reason for some results that contradict previously reports. For example, the calcification of nodules and osteogenic differentiation in porcine VIC cultures has been critically discussed, and no evidence has supported the calcification of porcine VIC cultures after 21 days in induction medium containing ascorbic acid, dexamethasone and β-glycerophosphate. Instead, the cells expressed a collagenous ECM and were activated towards a myofibroblastic phenotype [50]. By contrast, the human VIC preparations used in the present study showed clear calcification in the culture plates in response to ADGM, and this was confirmed by photometric detection of calcium ions in quantitative analysis, byAlizarin red and von Kossa staining of fixed cell culture pellets and initially by IR spectroscopy.

## Conclusions

Both active β-catenin and Sox9 are downregulated in calcifying VICs and upregulated in VICs co-treated with XAV-939. Therefore, discovery of the regulatory molecular processes of this human VIC model will require evaluation of alternative regulatory models (e.g. Notch signalling) as a next step. In addition, a need to identify the actual cell culture subpopulations that are responsible for the calcification process is envisioned. XAV-939 is able to prevent the *in vitro* calcification of VICs induced by ADGM and is therefore a substance of interest, on the one hand, for the investigation of relevant molecular pathways involved in osteogenesis and, on the other hand, as a small molecule inhibitor that can be implemented in *in vitro* and *in vivo* analyses for further drug development.

## Supporting information

S1 Fig MTT-based cell viability of VICs treated with 10, 1 and 0.1 μM XAV-939 for 72h. MTT-assay cell viability was not statistically significant altered after XAV-939 inhibitor incubation of VICs at different concentrations and an incubation time of 72h.
(TIF)

S2 Fig Histological analysis of the cellular pellet of a human VIC culture in DMEM without induction of calcification. Equivalent to Fig 2, A) shows a section stained with Movats Pentachrom B) Alizarin red and C) a combined von Kossa and HE staining, revealing no calcific mineralization in this condition.
(TIF)

S3 Fig Datasets of the study according to Figure number. (PDF)

## References

[1] Lindman BR, Clavel MA, Mathieu P, Iung B, Lancellotti P, Otto CM, et al Calcific aortic stenosis. Nat Rev Dis Primers. 2016;2:16006 10.1038/nrdp.2016.6 ; PubMed Central PMCID: PMCPMC5127286. 27188578 10.1038/nrdp.2016.6 PMC5127286

[2] Pilgrim T. WP. Aortenklappenersatz bei älteren Patienten mit schwerer Aortenstenose. Cardiovascular Medicine. 2010;13(6):197–203.

[3] Coffey S, Cairns BJ, Iung B. The modern epidemiology of heart valve disease. Heart. 2016;102(1):75–85. 10.1136/heartjnl-2014-307020 . 26541169 10.1136/heartjnl-2014-307020

[4] Hutcheson JD, Aikawa E, Merryman WD. Potential drug targets for calcific aortic valve disease. Nat Rev Cardiol. 2014;11(4):218–31. 10.1038/nrcardio.2014.1 ; PubMed Central PMCID: PMC4263317. 24445487 10.1038/nrcardio.2014.1 PMC4263317

[5] Rajamannan NM. Osteocardiology: Defining the Go/No-Go Time Point for Therapy. Cardiology. 2018;139(3):175–83. Epub 2018/02/03. 10.1159/000485074 . 29393145 10.1159/000485074

[6] Teo KK, Corsi DJ, Tam JW, Dumesnil JG, Chan KL. Lipid lowering on progression of mild to moderate aortic stenosis: meta-analysis of the randomized placebo-controlled clinical trials on 2344 patients. Can J Cardiol. 2011;27(6):800–8. 10.1016/j.cjca.2011.03.012 . 21742465 10.1016/j.cjca.2011.03.012

[7] Rajamannan NM, Evans FJ, Aikawa E, Grande-Allen KJ, Demer LL, Heistad DD, et al Calcific aortic valve disease: not simply a degenerative process: A review and agenda for research from the National Heart and Lung and Blood Institute Aortic Stenosis Working Group. Executive summary: Calcific aortic valve disease-2011 update. Circulation. 2011;124(16):1783–91. 10.1161/CIRCULATIONAHA.110.006767 ; PubMed Central PMCID: PMC3306614. 22007101 10.1161/CIRCULATIONAHA.110.006767 PMC3306614

[8] Leopold JA. Cellular mechanisms of aortic valve calcification. Circ Cardiovasc Interv. 2012;5(4):605–14. 10.1161/CIRCINTERVENTIONS.112.971028 ; PubMed Central PMCID: PMC3427002. 22896576 10.1161/CIRCINTERVENTIONS.112.971028 PMC3427002

[9] Liu X, Xu Z. Osteogenesis in calcified aortic valve disease: From histopathological observation towards molecular understanding. Prog Biophys Mol Biol. 2016;122(2):156–61. 10.1016/j.pbiomolbio.2016.02.002 . 26971958 10.1016/j.pbiomolbio.2016.02.002

[10] Liu AC, Joag VR, Gotlieb AI. The emerging role of valve interstitial cell phenotypes in regulating heart valve pathobiology. Am J Pathol. 2007;171(5):1407–18. 10.2353/ajpath.2007.070251 ; PubMed Central PMCID: PMCPMC2043503. 17823281 10.2353/ajpath.2007.070251 PMC2043503

[11] Chen JH, Yip CY, Sone ED, Simmons CA. Identification and characterization of aortic valve mesenchymal progenitor cells with robust osteogenic calcification potential. Am J Pathol. 2009;174(3):1109–19. 10.2353/ajpath.2009.080750 ; PubMed Central PMCID: PMCPMC2665769. 19218344 10.2353/ajpath.2009.080750 PMC2665769

[12] Langenbach F, Handschel J. Effects of dexamethasone, ascorbic acid and beta-glycerophosphate on the osteogenic differentiation of stem cells in vitro. Stem Cell Res Ther. 2013;4(5):117 10.1186/scrt328 ; PubMed Central PMCID: PMCPMC3854789. 24073831 10.1186/scrt328 PMC3854789

[13] Rutkovskiy A, Stenslokken KO, Vaage IJ. Osteoblast Differentiation at a Glance. Med Sci Monit Basic Res. 2016;22:95–106. doi: 10.12659/MSMBR.901142 ; PubMed Central PMCID: PMCPMC5040224. 27667570 10.12659/MSMBR.901142 PMC5040224

[14] Gu GJ, Chen T, Zhou HM, Sun KX, Li J. Role of Wnt/beta-catenin signaling pathway in the mechanism of calcification of aortic valve. J Huazhong Univ Sci Technolog Med Sci. 2014;34(1):33–6. 10.1007/s11596-014-1228-x . 24496676 10.1007/s11596-014-1228-x

[15] Rajamannan NM, Subramaniam M, Caira F, Stock SR, Spelsberg TC. Atorvastatin inhibits hypercholesterolemia-induced calcification in the aortic valves via the Lrp5 receptor pathway. Circulation. 2005;112(9 Suppl):I229–34. 10.1161/01.CIRCULATIONAHA.104.524306 ; PubMed Central PMCID: PMCPMC3951868. 16159822 10.1161/01.CIRCULATIONAHA.104.524306 PMC3951868

[16] Fang M, Alfieri CM, Hulin A, Conway SJ, Yutzey KE. Loss of beta-catenin promotes chondrogenic differentiation of aortic valve interstitial cells. Arterioscler Thromb Vasc Biol. 2014;34(12):2601–8. 10.1161/ATVBAHA.114.304579 ; PubMed Central PMCID: PMCPMC4239156. 25341799 10.1161/ATVBAHA.114.304579 PMC4239156

[17] Caira FC, Stock SR, Gleason TG, McGee EC, Huang J, Bonow RO, et al Human degenerative valve disease is associated with up-regulation of low-density lipoprotein receptor-related protein 5 receptor-mediated bone formation. J Am Coll Cardiol. 2006;47(8):1707–12. 10.1016/j.jacc.2006.02.040 ; PubMed Central PMCID: PMCPMC3951851. 16631011 10.1016/j.jacc.2006.02.040 PMC3951851

[18] Rajamannan NM. The role of Lrp5/6 in cardiac valve disease: experimental hypercholesterolemia in the ApoE-/- /Lrp5-/- mice. J Cell Biochem. 2011;112(10):2987–91. 10.1002/jcb.23221 ; PubMed Central PMCID: PMCPMC3263342. 21678468 10.1002/jcb.23221 PMC3263342

[19] Rajamannan NM. TIEG1 is upregulated in Lrp5/6-mediated valve osteogenesis. J Cell Biochem. 2018 10.1002/jcb.27606 . 30246479 10.1002/jcb.27606 PMC6336497

[20] Chen JH, Chen WL, Sider KL, Yip CY, Simmons CA. beta-catenin mediates mechanically regulated, transforming growth factor-beta1-induced myofibroblast differentiation of aortic valve interstitial cells. Arterioscler Thromb Vasc Biol. 2011;31(3):590–7. 10.1161/ATVBAHA.110.220061 . 21127288 10.1161/ATVBAHA.110.220061

[21] Gao X, Zhang L, Gu G, Wu PH, Jin S, Hu W, et al The effect of oxLDL on aortic valve calcification via the Wnt/ beta-catenin signaling pathway: an important molecular mechanism. J Heart Valve Dis. 2015;24(2):190–6. . 26204684

[22] Xie C, Shen Y, Hu W, Chen Z, Li Y. Angiotensin II promotes an osteoblast-like phenotype in porcine aortic valve myofibroblasts. Aging Clin Exp Res. 2016;28(2):181–7. 10.1007/s40520-015-0408-2 . 26197716 10.1007/s40520-015-0408-2

[23] Grigoryan T, Wend P, Klaus A, Birchmeier W. Deciphering the function of canonical Wnt signals in development and disease: conditional loss- and gain-of-function mutations of beta-catenin in mice. Genes Dev. 2008;22(17):2308–41. 10.1101/gad.1686208 ; PubMed Central PMCID: PMCPMC2749675. 18765787 10.1101/gad.1686208 PMC2749675

[24] Peacock JD, Levay AK, Gillaspie DB, Tao G, Lincoln J. Reduced sox9 function promotes heart valve calcification phenotypes in vivo. Circ Res. 2010;106(4):712–9. 10.1161/CIRCRESAHA.109.213702 ; PubMed Central PMCID: PMCPMC2863131. 20056916 10.1161/CIRCRESAHA.109.213702 PMC2863131

[25] Peacock JD, Huk DJ, Ediriweera HN, Lincoln J. Sox9 transcriptionally represses Spp1 to prevent matrix mineralization in maturing heart valves and chondrocytes. PLoS One. 2011;6(10):e26769 10.1371/journal.pone.0026769 ; PubMed Central PMCID: PMCPMC3202586. 22046352 10.1371/journal.pone.0026769 PMC3202586

[26] Li J, Dong S. The Signaling Pathways Involved in Chondrocyte Differentiation and Hypertrophic Differentiation. Stem Cells Int. 2016;2016:2470351 10.1155/2016/2470351 ; PubMed Central PMCID: PMCPMC5198191 publication of this paper. 28074096 10.1155/2016/2470351 PMC5198191

[27] Hill TP, Spater D, Taketo MM, Birchmeier W, Hartmann C. Canonical Wnt/beta-catenin signaling prevents osteoblasts from differentiating into chondrocytes. Dev Cell. 2005;8(5):727–38. 10.1016/j.devcel.2005.02.013 . 15866163 10.1016/j.devcel.2005.02.013

[28] Huang SM, Mishina YM, Liu S, Cheung A, Stegmeier F, Michaud GA, et al Tankyrase inhibition stabilizes axin and antagonizes Wnt signalling. Nature. 2009;461(7264):614–20. 10.1038/nature08356 . 19759537 10.1038/nature08356

[29] Tortelote GG, Reis RR, de Almeida Mendes F, Abreu JG. Complexity of the Wnt/betacatenin pathway: Searching for an activation model. Cell Signal. 2017;40:30–43. 10.1016/j.cellsig.2017.08.008 . 28844868 10.1016/j.cellsig.2017.08.008

[30] Hulin A, Moore V, James JM, Yutzey KE. Loss of Axin2 results in impaired heart valve maturation and subsequent myxomatous valve disease. Cardiovasc Res. 2017;113(1):40–51. 10.1093/cvr/cvw229 ; PubMed Central PMCID: PMCPMC5220675. 28069701 10.1093/cvr/cvw229 PMC5220675

[31] Huk DJ, Austin BF, Horne TE, Hinton RB, Ray WC, Heistad DD, et al Valve Endothelial Cell-Derived Tgfbeta1 Signaling Promotes Nuclear Localization of Sox9 in Interstitial Cells Associated With Attenuated Calcification. Arterioscler Thromb Vasc Biol. 2016;36(2):328–38. 10.1161/ATVBAHA.115.306091 ; PubMed Central PMCID: PMCPMC4732913. 26634652 10.1161/ATVBAHA.115.306091 PMC4732913

[32] Wirrig EE, Yutzey KE. Conserved transcriptional regulatory mechanisms in aortic valve development and disease. Arterioscler Thromb Vasc Biol. 2014;34(4):737–41. 10.1161/ATVBAHA.113.302071 ; PubMed Central PMCID: PMCPMC3967128. 24665126 10.1161/ATVBAHA.113.302071 PMC3967128

[33] Acharya A, Hans CP, Koenig SN, Nichols HA, Galindo CL, Garner HR, et al Inhibitory role of Notch1 in calcific aortic valve disease. PLoS One. 2011;6(11):e27743 10.1371/journal.pone.0027743 ; PubMed Central PMCID: PMCPMC3218038. 22110751 10.1371/journal.pone.0027743 PMC3218038

[34] Witt W, Buttner P, Jannasch A, Matschke K, Waldow T. Reversal of myofibroblastic activation by polyunsaturated fatty acids in valvular interstitial cells from aortic valves. Role of RhoA/G-actin/MRTF signalling. J Mol Cell Cardiol. 2014;74:127–38. 10.1016/j.yjmcc.2014.05.008 . 24839911 10.1016/j.yjmcc.2014.05.008

[35] Dritsa V, Pissaridi K, Koutoulakis E, Mamarelis I, Kotoulas C, Anastassopoulou J. An infrared spectroscopic study of aortic valve. A possible mechanism of calcification and the role of magnesium salts. In Vivo. 2014;28(1):91–8. . 24425841

[36] Mangialardo S, Cottignoli V, Cavarretta E, Salvador L, Postorino P, Maras A. Pathological biominerals: Raman and infrared studies of bioapatite deposits in human heart valves. Appl Spectrosc. 2012;66(10):1121–7. 10.1366/12-06606 . 23031694 10.1366/12-06606

[37] Jastrzebska M, Zalewska-Rejdak J, Mroz I, Barwinski B, Wrzalik R, Kocot A, et al Atomic force microscopy and FT-IR spectroscopy investigations of human heart valves. Gen Physiol Biophys. 2006;25(3):231–44. . 17197723

[38] Lefebvre V, Dvir-Ginzberg M. SOX9 and the many facets of its regulation in the chondrocyte lineage. Connect Tissue Res. 2017;58(1):2–14. 10.1080/03008207.2016.1183667 ; PubMed Central PMCID: PMCPMC5287363. 27128146 10.1080/03008207.2016.1183667 PMC5287363

[39] Zhang X, Hao J. Development of anticancer agents targeting the Wnt/beta-catenin signaling. Am J Cancer Res. 2015;5(8):2344–60. ; PubMed Central PMCID: PMCPMC4568771. 26396911 PMC4568771

[40] Mallinger A, Crumpler S, Pichowicz M, Waalboer D, Stubbs M, Adeniji-Popoola O, et al Discovery of potent, orally bioavailable, small-molecule inhibitors of WNT signaling from a cell-based pathway screen. J Med Chem. 2015;58(4):1717–35. 10.1021/jm501436m ; PubMed Central PMCID: PMCPMC4767141. 25680029 10.1021/jm501436m PMC4767141

[41] Lehtio L, Chi NW, Krauss S. Tankyrases as drug targets. FEBS J. 2013;280(15):3576–93. 10.1111/febs.12320 . 23648170 10.1111/febs.12320

[42] James RG, Davidson KC, Bosch KA, Biechele TL, Robin NC, Taylor RJ, et al WIKI4, a novel inhibitor of tankyrase and Wnt/ss-catenin signaling. PLoS One. 2012;7(12):e50457 10.1371/journal.pone.0050457 ; PubMed Central PMCID: PMCPMC3515623. 23227175 10.1371/journal.pone.0050457 PMC3515623

[43] Wang Q, Zang W, Han L, Yang L, Ye S, Ouyang J, et al Wenyang Huazhuo Tongluo formula inhibits fibrosis via suppressing Wnt/beta-catenin signaling pathway in a Bleomycin-induced systemic sclerosis mouse model. Chin Med. 2018;13:17 10.1186/s13020-018-0175-z ; PubMed Central PMCID: PMCPMC5870182. 29599817 10.1186/s13020-018-0175-z PMC5870182

[44] Mathieu P, Boulanger MC. Basic mechanisms of calcific aortic valve disease. Can J Cardiol. 2014;30(9):982–93. 10.1016/j.cjca.2014.03.029 . 25085215 10.1016/j.cjca.2014.03.029

[45] Latif N, Sarathchandra P, Chester AH, Yacoub MH. Expression of smooth muscle cell markers and co-activators in calcified aortic valves. Eur Heart J. 2015;36(21):1335–45. 10.1093/eurheartj/eht547 . 24419809 10.1093/eurheartj/eht547

[46] Louvet L, Bazin D, Buchel J, Steppan S, Passlick-Deetjen J, Massy ZA. Characterisation of calcium phosphate crystals on calcified human aortic vascular smooth muscle cells and potential role of magnesium. PLoS One. 2015;10(1):e0115342 10.1371/journal.pone.0115342 ; PubMed Central PMCID: PMCPMC4301909. 25607936 10.1371/journal.pone.0115342 PMC4301909

[47] Iyemere VP, Proudfoot D, Weissberg PL, Shanahan CM. Vascular smooth muscle cell phenotypic plasticity and the regulation of vascular calcification. J Intern Med. 2006;260(3):192–210. 10.1111/j.1365-2796.2006.01692.x . 16918817 10.1111/j.1365-2796.2006.01692.x

[48] Shroff RC, Shanahan CM. The vascular biology of calcification. Semin Dial. 2007;20(2):103–9. 10.1111/j.1525-139X.2007.00255.x . 17374082 10.1111/j.1525-139X.2007.00255.x

[49] Pal SN, Golledge J. Osteo-progenitors in vascular calcification: a circulating cell theory. J Atheroscler Thromb. 2011;18(7):551–9. . 21551961 10.5551/jat.8656

[50] Cloyd KL, El-Hamamsy I, Boonrungsiman S, Hedegaard M, Gentleman E, Sarathchandra P, et al Characterization of porcine aortic valvular interstitial cell 'calcified' nodules. PLoS One. 2012;7(10):e48154 10.1371/journal.pone.0048154 ; PubMed Central PMCID: PMCPMC3482191. 23110195 10.1371/journal.pone.0048154 PMC3482191

[51] Bowler MA, Merryman WD. In vitro models of aortic valve calcification: solidifying a system. Cardiovasc Pathol. 2015;24(1):1–10. 10.1016/j.carpath.2014.08.003 ; PubMed Central PMCID: PMCPMC4268061. 25249188 10.1016/j.carpath.2014.08.003 PMC4268061

