# Supplementary figures and images for: Treatment with XAV-939 prevents in vitro calcification of human valvular interstitial cells
Source: PLoS One. 2018 Dec 7;13(12):e0208774. doi: 10.1371/journal.pone.0208774 (PMC6286025; doi:10.1371/journal.pone.0208774)

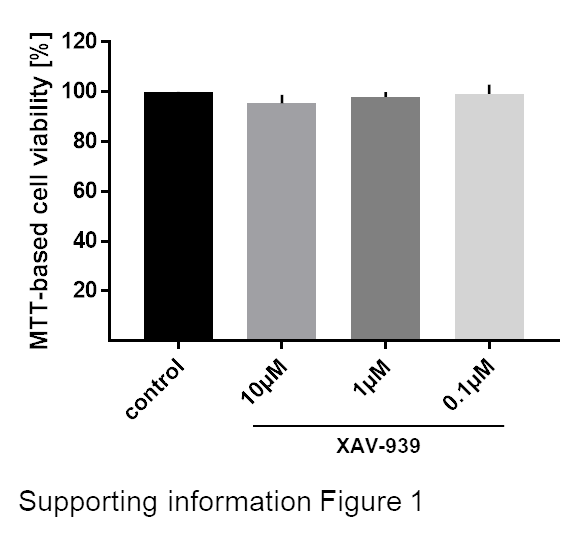

Supplement: S1 Fig — MTT-assay cell viability was not statistically significant altered after XAV-939 inhibitor incubation of VICs at different concentrations and an incubation time of 72h. (TIF) [file pone.0208774.s001.tif]

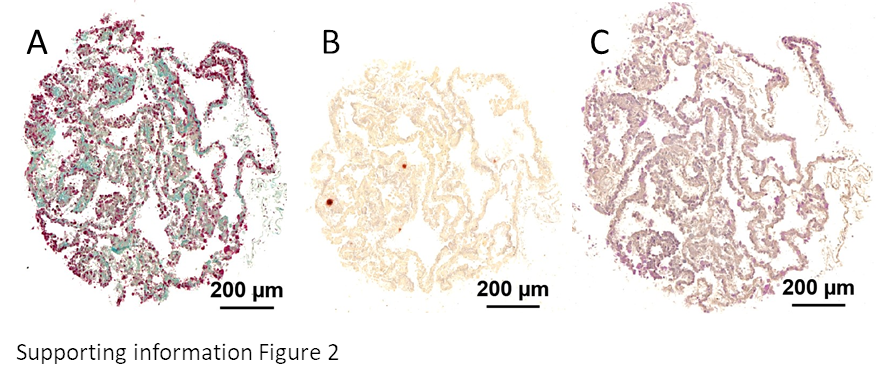

Supplement: S2 Fig — Equivalent to Fig 2, A) shows a section stained with Movats Pentachrom B) Alizarin red and C) a combined von Kossa and HE staining, revealing no calcific mineralization in this condition. (TIF) [file pone.0208774.s002.tif]
