# Supplementary material for: Treatment with XAV-939 prevents in vitro calcification of human valvular interstitial cells
Source: PLoS One. 2018 Dec 7;13(12):e0208774. doi: 10.1371/journal.pone.0208774 (PMC6286025; doi:10.1371/journal.pone.0208774)
Supplement: S3 Fig — (PDF) [file pone.0208774.s003.pdf]

S3 Fig:

Data, manuscript „Treatment with XAV-939 prevents *in vitro* calcification of human valvular interstitial cells"; Dittfeld, Reimann et al

Ca2+ [mol/kg protein]; Figure 1

DMEM	ADGM
0,7682562	1,446046
0,1593062	1,076728
0,5270988	3,802261
0,2057632	0,4541912
0,1573437	0,2288075
0,09254184	0,2515981
0,09011128	2,519767
0,3036537	0,6980787
0,1116676	6,84017
0,2485094	0,347451
0,2383973	6,913989

Ca2+ [mol/kg protein]; Figure 4

DMEM	ADGM	DMEM XAV-939	ADGM XAV-939
0,383175	4,955254	0,424061	0,417237
0,092542	0,251598	0,165687	0,084033
0,09629	3,120502	0,309159	0,214398
0,303654	0,698079	0,61079	0,504464
0,111668	6,84017	0,22105	0,236345
0,248509	0,347451	0,556334	0,247713
0,238397	6,913989	0,704715	0,208393

Western Blot; Alkaline Phosphatase relative to GAPDH; Figure 5

DMEM	ADGM
0,0012	0,6002
0,0257	2,1461
0,0087	0,5558
0,0053	0,8725
5,02513E-05	0,5759

DMEM XAV-939	ADGM XAV-939
0,0031	0,3633681
0,0201	1,30668
0,002769231	0,3767415
0,0167	0,5445091
0,0033	0,188961

Western Blot; Axin 1 relative to GAPDH, Figure 6

DMEM	ADGM	DMEM XAV-939	ADGM XAV-939
0,4983	0,1605	1,0709	1,4302
0,4763	0,2219	0,7115	0,8441
0,4363	0,082	1,0073	1,832

Western Blot; β-Catenin relative to GAPDH, Figure 6

DMEM	ADGM	DMEM XAV-939	ADGM XAV-939
2,5625	1,5294	1,4132	1,222035
2,019	1,9047	1,7433	1,6268
1,2017	3,4821	2,469	2,62392
1,7236	1,6431	3,3313	2,492262
3,0316	5,8584	2,6739	2,5896

Western Blot; Non-Phospho-β-Catenin relative to GAPDH, Figure 6

0,126694	0,1070285	0,3154362	0,6198628
0,07831978	0,03408068	0,09150327	0,2466785
0,2288889	0,2088992	0,2009709	0,327423
0,3060345	0,1057618	0,4592	0,5985027
0,1651568	0,03386314	0,1776119	0,7104221

Western Blot; SOX 9 relative to GAPDH, Figure 6

DMEM	ADGM	DMEM XAV-939	ADGM XAV-939
0,3498	0,1348	1,3653	0,5181802
2,2229	0,522	8,2947	1,630713
0,1594	0,4027	0,8009	0,712363
0,309	0,2106	1,0136	0,7056733

SOX 9 nuclear localization rate [%], Figure 7

DMEM	ADGM
6,3	3,2
31,1	10,5
22,9	1,2
43,8	11,8
56,5	29,1

DMEM XAV-939	ADGM XAV-939
41,5	41,6
38,6	26,6
11,1	17,7
46,3	7,6
66,2	36,8
